# Urinary Transthyretin as a Biomarker in ATTRv Val50Met Amyloidosis

**DOI:** 10.3390/pathophysiology29030025

**Published:** 2022-06-29

**Authors:** Hiroaki Matsushita, Yohei Misumi, Teruaki Masuda, Masamitsu Okada, Fumika Inoue, Mitsuharu Ueda, Yukio Ando

**Affiliations:** 1 Department of Amyloidosis Research, Faculty of Pharmaceutical Sciences, Nagasaki International University, 2825-7 Huis Ten Bosch, Nagasaki 859-3298, Japan; pladofam@gmail.com (F.I.); andoy430@niu.ac.jp (Y.A.); 2 Department of Neurology, Graduate School of Medical Sciences, Kumamoto University, 1-1-1 Honjo, Kumamoto 860-0811, Japan; misumiyohei@hotmail.co.jp (Y.M.); t.masuda0903@gmail.com (T.M.); masamitsu.dreacome@gmail.com (M.O.); mitt@rb3.so-net.ne.jp (M.U.)

**Keywords:** amyloidosis, ATTR, biomarker, transthyretin, urine

## Abstract

Transthyretin (TTR), the precursor protein for amyloidogenic TTR (ATTR) amyloidosis, forms tetramers and escapes glomerular filtration by binding with thyroxine and retinol-binding protein. However, variant TTRs are unstable as tetramers, so monomeric TTR has become the precursor protein of amyloid deposits, via protein misfolding. The aim of the study was to evaluate the utility of urinary TTR in the diagnosis of ATTRv amyloidosis. Urinary samples from healthy volunteers, ATTRv V50M amyloidosis patients, and asymptomatic carriers of the ATTRv V50M gene were analysed using ELISA. To analyse the different forms of TTR secreted to the urine, we performed Western blotting and mass spectrometry. Urinary TTR concentrations were significantly higher in the ATTRv V50M amyloidosis patients than they were in the healthy volunteers and asymptomatic carriers of the gene. Although the TTR concentrations were negligible in the healthy volunteers, they were correlated with disease progression and urinary albumin concentrations in the ATTRv V50M amyloidosis patients. The Western blotting and mass spectrometry revealed the presence of monomeric wild-type and variant TTRs in the urine. Urinary TTR concentrations may become a more sensitive biomarker of ATTRv progression than albumin.

## 1. Introduction

Variant transthyretin amyloid (ATTRv) amyloidosis is a hereditary, life-threatening, and under-recognized disease, in which a misfolded transthyretin (TTR) protein forms amyloid fibrils and deposits in organs and tissues, thereby disrupting normal organ function and tissue structure [1]. In this disease, amyloid deposition occurs in various organs and tissues, such as peripheral nerves, the heart, the kidney, the intestine, various glands, the autonomic nervous system, ocular tissues, and the bladder and urinary tract [2,3,4,5,6]. Of these tissues, kidney dysfunction sometimes occurs at the early stage of the disease. Research has also focused on age-related sporadic amyloidosis, induced by wild-type TTR (ATTRwt) [7,8]. Cardiac amyloidosis and orthopaedic-related amyloidosis are the main clinical manifestations of ATTRwt amyloidosis [9,10,11]. In both types of ATTR amyloidosis, ATTR amyloid deposition has sometimes been reported in the urinary organs [12]. The reason why the bladder becomes a target organ in ATTR amyloidosis is not well understood.

In aqueous humour and cerebrospinal fluid, significant concentrations of TTR are detected because the retinal pigments epithelium and choroid plexus produce TTR [13]. In addition, we have confirmed the presence of TTR in saliva. These findings suggest that TTR is present in every body fluid, including urine, but the role of TTR in these fluids is not clear.

Mass spectrometric analyses revealed that, in addition to unmodified TTR, the cysteine (Cys)-conjugated form and the oxidized form of TTR is predominantly detected in plasma. In ATTRv amyloidosis and ATTRwt, a variant form of TTR was detected by the analyses. In the amyloid formation mechanism, since oxidative stress is deeply connected with amyloid fibril formation, the post-translational modification of the amyloid precursor protein may play an important role in the initiation or propagation of amyloidosis.

Since monomeric TTR is a 14-kDa protein, it easily undergoes glomerular filtration if it does not form tetramers [14]. Monomeric or tetrameric forms of TTR may be excreted into urine in some pathological conditions. TTR amyloid deposition has been well documented as occurring frequently in the urinary tract, including the bladder, in ATTRv amyloidosis. In other studies, histopathologic analyses revealed that, in ATTRv amyloidosis, significant amyloid deposition was observed in perivascular lesions and bladder stroma [15,16]. Moreover, amyloidomas that derived from ATTRwt were sometimes found [17]. The presence of TTR in urine may, thus, play an important role in amyloid formation if significant amounts of TTR are excreted into urine.

In this study, we attempted to detect urinary TTR and to analyse the relationship between different forms of TTR and TTR amyloid deposition by means of biochemical methods, and the possible diagnostic importance of this is discussed.

## 2. Patients and Methods

### 2.1. Patients

Urine samples were obtained from 15 healthy volunteers (8 men, aged 26.4 ± 4.5 years; and 7 women, aged 27.7 ± 5.6 years) and from 17 patients with ATTRv V50M amyloidosis and from 4 asymptomatic gene carriers of ATTRv V50M (10 men, aged 37.2 ± 5.2 years; and 11 women, aged 43.5 ± 10.2 years). Precise clinical information of healthy volunteers and ATTRv V50M are presented in Table 1. All these samples were analysed biochemically and compared with those obtained from the 15 healthy volunteers. Twelve ATTRv Val50Met patients who had undergone liver transplants were included in this study. The patients and healthy volunteers with inflammation-evaluated WBC and CRP levels were eliminated from this study. We used the clinical Kumamoto score, which is widely used throughout the world, as a clinical score to evaluate the type and number of clinical manifestations of ATTRv amyloidosis [15].

### 2.2. Analysis of TTR Concentrations in Urine Samples

Urine concentrations of TTR were analysed by using the ELISA. A hundred µL urine samples, collected over 24 h, were used for the analysis. Briefly, 100 µL of a sheep anti-human TTR antibody (Biogenesis, England), at a 1:7000 dilution, was added to each well and incubated at 4 °C overnight, after which 250 μL of a blocking solution was added. As a primary antibody, 100 µL of a rabbit anti-human TTR antibody (DAKO, Glostrup, Denmark), diluted 1:10,000, was added to each well, and the reaction continued for 1 h at room temperature. Next, a secondary antibody—100 µL of HRP-labelled goat anti-rabbit IgG antibody (DAKO) diluted 1:5000—was added, and the mixture was reacted at room temperature for 1 h. Finally, 100 μL of TMB Microwell Peroxidase Substrate (Kirkegaard & Perry Laboratories, Gaithersburg, MD, USA) was added to each sample, and absorbance of the samples was measured at the wavelength of 450 nm. A standard curve was drawn using 4 different concentrations of recombinant TTRwt samples. The assay was performed three times.

### 2.3. Analysis of Urine Samples by Using Matrix-Assisted Laser Desorption/Ionization–Time-of-Flight Mass Spectrometry (MALDI-TOF MS)

Urine samples (each 1 mL) were dialysed against distilled water using a dialysis membrane (Spectra/Por 7 Membrane, MWCO = 3.5 kD, Funakoshi, Tokyo, Japan). Lyophilisation of the solution was performed using a vacuum freeze dryer (FTS System Inc., New York, NY, USA) and the sample was dissolved in saline. To remove albumin, the solution was incubated for 2 days at 4 °C, with 30 μL of a rabbit polyclonal anti-albumin antibody (INTER-CELL TECHNOLOGIES Inc., Hopewell, VA, USA). After the mixture was centrifuged at 10,000 rpm for 15 min, the supernatant was collected and incubated for 2 days at 4 °C, with 30 μL of a rabbit polyclonal anti-TTR antibody (A0002; Agilent, Santa Clara, CA, USA). After the mixture was centrifuged at 10,000 rpm for 15 min, the pellet was washed twice with 300 μL of saline and 300 μL of distilled water. The precipitates were dissolved in 20 μL of 4% acetic acid and 4% acetonitrile in distilled water, as described previously [18]. The matrix was a saturated solution of sinapinic acid (SPA) in 1:2 acetonitrile: water (*v*/*v*), containing 0.1% TFA. After samples were mixed with SPA, the dried mixtures on the plate were analysed via MALDI-TOF MS (Bruker, Bremen, Germany).

### 2.4. Analysis of Serum Samples by Using MALDI-TOF MS

Serum TTR was analysed by using MALDI-TOF MS, as previously described [18]. In brief, 50 μL of serum was mixed with 20 μL of an anti-TTR antibody (Agilent). The precipitate was centrifuged at 9000× *g* for 5 min and washed twice with 100 μL of saline and 100 μL of distilled water. The pellet was dissolved in 50 μL of 4% acetonitrile and 4% acetic acid in distilled water, and this solution was filtered through a 1000-kDa centrifugal concentrator (Pall Filtron, Northborough, MA, USA). After samples were mixed with SPA, the dried mixtures on the plate were analysed via MALDI-TOF MS.

### 2.5. Immunoblot Analysis

Western blotting was performed as previously described [19]. Samples of urine were separated by means of non-reducing sodium dodecyl sulfate–polyacrylamide gel electrophoresis and were then transferred onto polyvinylidene difluoride membranes. The membranes were blocked with 2.5% skim milk in 1 × Tris-buffered saline with Tween 20 (TBST), for 1 h at room temperature. Next, the membranes were washed three times with 0.2% skim milk in 1 × TBST for 5 min and incubated overnight at 4 °C, with a rabbit polyclonal antibody, against TTR at a 1:1500 dilution. After the membranes were washed, they were incubated with a secondary antibody, conjugated with HRP (anti-rabbit IgG for anti-TTR, at a 1:4000 dilution) (Sigma-Aldrich, Saint Louis, MO, USA) for 1 h at room temperature. Western blot bands were detected via the enhanced chemiluminescence technique, with the ECL prime detection kit (GE Healthcare, Little Chalfont, Buckinghamshire, UK).

### 2.6. Statistics

We used Student’s *t*-test or the Mann–Whitney U-test to identify significant differences between two conditions. *p*-values less than 0.01 or 0.05 were considered to be significant. Data are shown as means + SD. Correlations between each term were analysed by using Pearson’s correlation coefficient. Data were analysed by using Statcel-the Useful Addin Forms on Excel (OMS, Tokyo, Japan).

## 3. Results

### 3.1. Urinary TTR in Patients with ATTRv V50M Amyloidosis

**Figure 1:**
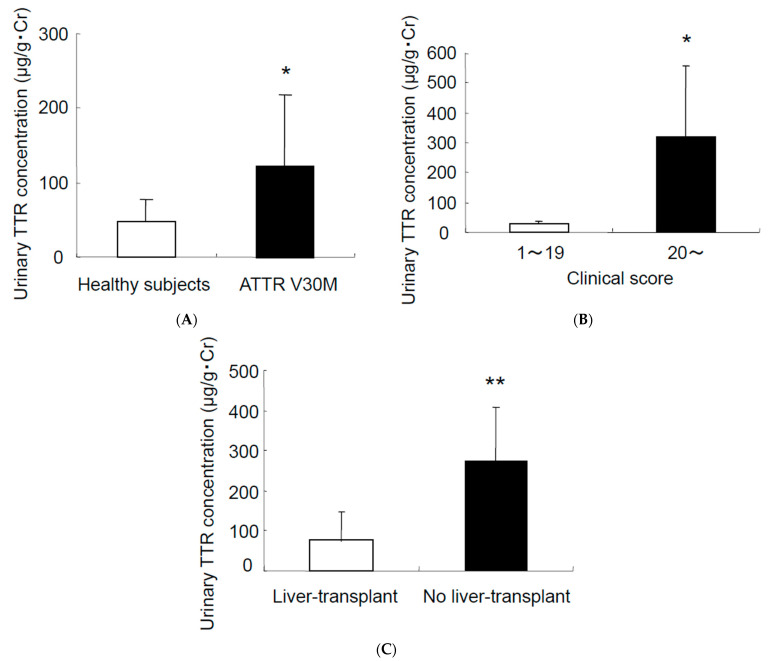
Urinary TTR concentrations in patients with ATTRv V50M amyloidosis. (**A**) Urinary TTR concentrations in healthy subjects (*n* = 15) and in patients with ATTRv V50M amyloidosis (*n* = 17). (**B**) Urinary TTR concentrations plotted against ATTRv score (1–19: *n* = 10, and 20: < *n* = 7). (**C**) Urinary TTR concentrations in patients with ATTRv V50M (*n* = 12) who had liver transplants and patients with ATTRv V50M (*n* = 5) but no liver transplants; * *p* < 0.05; ** *p* < 0.01. Data represent means + SD.

Urinary TTR concentrations were much higher in the patients with ATTRv V50M than they were in the healthy volunteers (Figure 1A). The amounts of urinary TTR increased as the disease progressed (Figure 1B). The urinary TTR concentrations in patients who had undergone liver transplants were much lower than those in patients who had not undergone liver transplants (Figure 1C). Although the urinary TTR concentrations of transplanted patients were slightly increased compared with those of the healthy volunteers, statistical significance was not observed between the healthy volunteers versus non-transplanted patients. This indicates that liver transplantation should have the effect of protecting kidney function. In our study, only three late-onset ATTRv V50M patients were included. Although we could not precisely compare the data with those of early-onset patients, the tendency was similar. 

### 3.2. Correlation between Urinary TTR Concentrations and Kidney Function Markers

**Figure 2:**
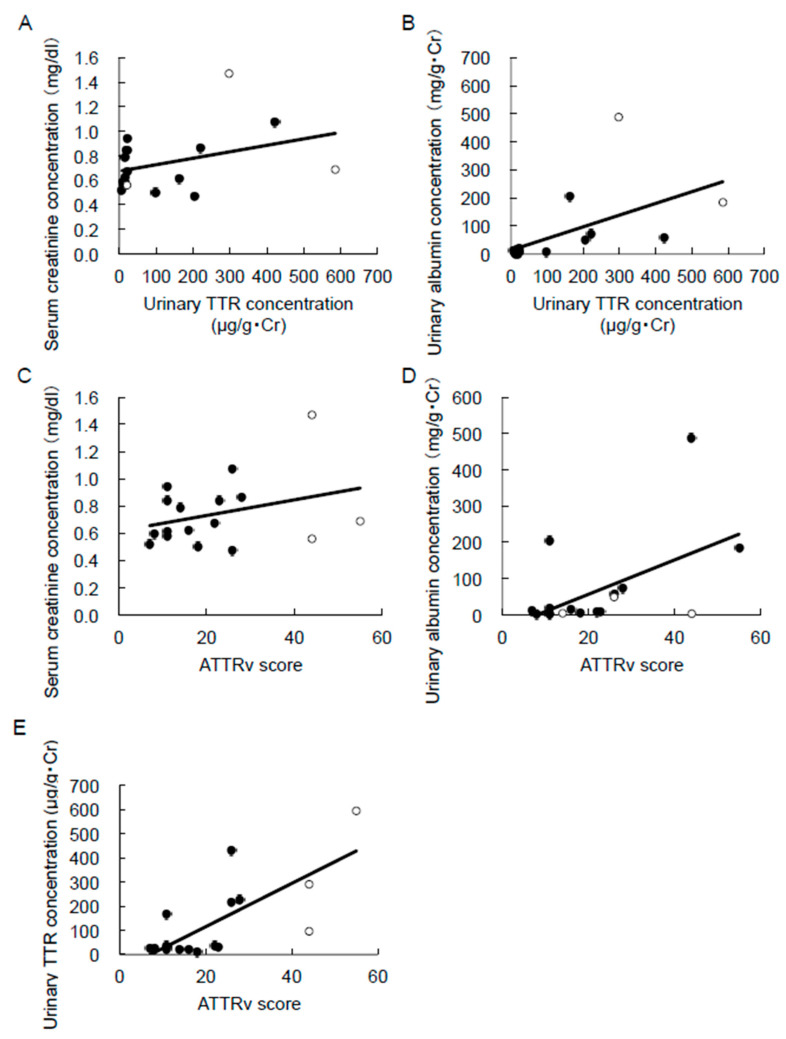
Correlation between urinary TTR concentrations and kidney function markers. (**A**) Serum creatinine concentration (*n* = 17, *r* = 0.358, *p* = 0.110), and (**B**) urinary albumin concentration (*n* = 17, *r* = 0.570, *p* < 0.016) versus urinary TTR concentrations of ATTRv. (**C**) Serum creatinine concentration (*n* = 17, *r* = 0.319, *p* = 0.211), (**D**) urinary albumin concentration (*n* = 17, *r* = 0.531, *p* < 0.028), (**E**) urinary TTR concentrations (*n* = 17, *r* = 0.741, *p* < 0.0006) versus the ATTRv score. The correlation coefficient (*r*) is shown. White circles—late onset.

Correlations between urinary TTR concentrations and other factors, including urinary albumin and creatinine concentrations were analysed (Figure 2): (A) the serum creatinine concentration versus the urinary TTR concentrations of ATTRv (*r* = 0.358, *p* = 0.110); (B) the urinary albumin concentrations versus the TTR concentrations of ATTRv (*r* = 0.570, *p* < 0.016); (C) the serum creatinine concentration versus the clinical ATTRv score (*r* = 0.319, *p* = 0.211); (D) the urinary albumin concentration versus the ATTRv score (*r* = 0.531, *p* < 0.028); and (E) the urinary TTR concentration versus the ATTRv score. The urinary TTR concentrations versus the ATTRv score (*r* = 0.741, *p* < 0.0006) had a much stronger correlation than that of albumin. The serum creatinine (Figure 2A,C), β_2_ microglobulin (β_2_M), and N-acetyl glucosamidase (NAG) concentrations versus the urinary TTR concentration showed no statistical significance (β_2_M: *r* = 0.215, *p* < 0.375; NAG: *r* = 0.087, *p* < 0.720). 

### 3.3. Biochemical Analyses for Urinary TTR

#### Western Blotting Analyses for Urinary TTR

**Figure 3:**
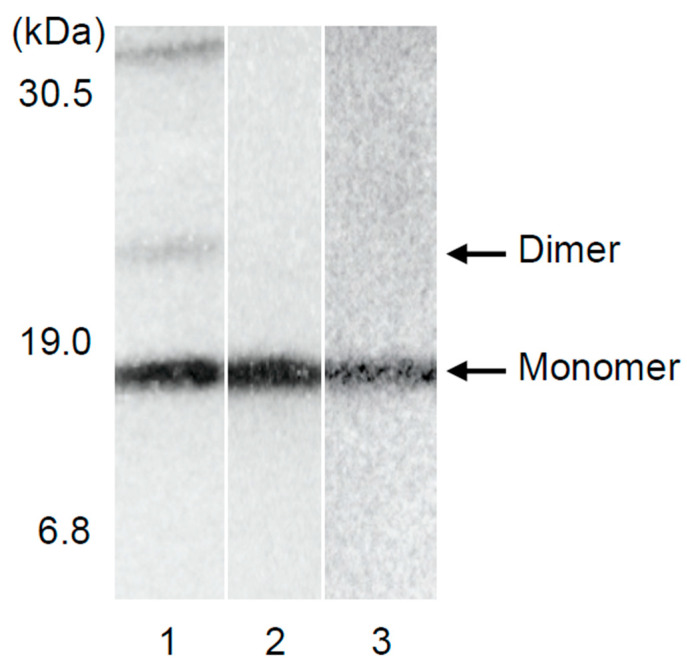
Western blotting of urinary TTR. Lane 1 shows serum from a healthy subject (No. 2 in Table 1), lane 2 shows urine from a healthy subject (No. 6 in Table 1), and lane 3 shows urine from an ATTRv V50M amyloidosis (No. 7 in Table 1). Representative samples are shown in the Figure.

To elucidate the types of TTR in urine, Western blotting was performed. Urine from the healthy volunteers and the ATTRv patients without renal impairment contained only trace amounts of monomeric TTR. Figure 3 shows the sample of a typical patient. Similar data were obtained in asymptomatic gene carriers and ATTRv patients without renal dysfunction (Figure 3).

### 3.4. Quantitative MS Analyses for Urinary TTR

**Figure 4:**
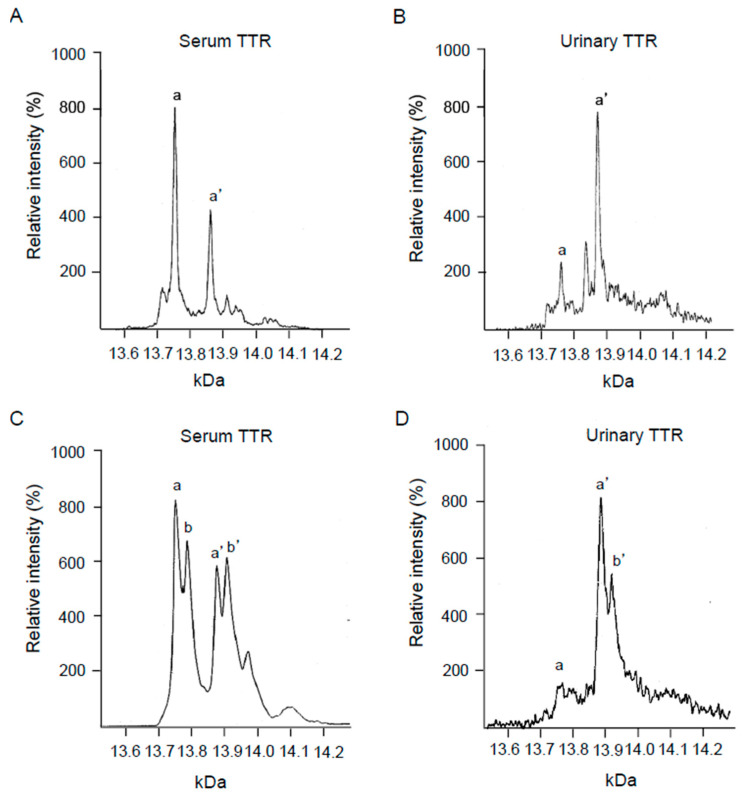
MS analysis for urinary TTR. (**A**) Serum from healthy subjects (No. 3 in Table 1), (**B**) urine from healthy subjects (No. 15 in Table 1), (**C**) serum from patients with ATTRv V50M amyloidosis (No. 10 in Table 1), and (**D**) urine from patients with ATTRv V50M amyloidosis (No. 11 in Table 1). a: normal TTR, a′: cysteine-binding normal TTR, b: ATTRv V50M, and b′: cysteine-binding ATTRv V50M.

To quantitatively analyse the urine and serum for TTR, mass spectrometry was utilised (Figure 4). As seen in Figure 4A, 13,761 Da and 13,880 Da peaks were detected as free forms of serum TTR and cysteine-conjugated forms of TTR. In Figure 4C, in addition to the TTRwt peaks, their increased peaks can be seen to indicate that ATTRv V50M were detected. Figure 4C demonstrated the serum TTR of the ATTRv V50M patient. Figure 4B,D, in addition to evidence for the presence of unmodified TTR in the urine, also shows that the cysteine-conjugated forms of TTRwt and ATTRv V50M were predominant. 

## 4. Discussion

In our study, we clearly demonstrated that the concentration of urinary TTR was higher in patients with ATTRv V50M than it was in the healthy volunteers (Figure 1). Although the urinary TTR concentrations of liver-transplanted patients were slightly increased compared with those of the healthy volunteers, statistical significance was not observed. This suggests that liver transplantation should have the effect of protecting kidney function. However, it is very important to note that liver-transplanted patients still have amyloid deposition in their tissues and clinical manifestations of ATTRv V50M. Therefore, the fact that total analyses using both transplanted and non-transplanted patients’ data for nephrotic function is worth reporting.

The urinary TTR concentrations did not correlate with the serum TTR concentrations, suggesting that nephrotic changes or changes in TTR molecule metabolism may induce this phenomenon. With regard to the clinical manifestations of patients with ATTRv, as analysed by using the clinical Kumamoto ATTRv score [20], patients with serious ATTRv had higher TTR concentrations in their urine, compared with the patients with mild ATTRv V50M (Figure 1). Negligible TTR concentrations were seen in the urine of the asymptomatic carriers of ATTRv amyloidosis. These results suggest that nephrotic impairment is generally correlated with amyloid deposition, and the measurement of TTR in urine may become a possible biomarker to aid in diagnosing ATTRv V50M amyloidosis and in determining the degree of its progression [21]. In addition, this phenomenon did not differ in the early- and late-onset ATTRv Val50Met patients. 

It should be noted that all the patients employed in this study were ATTRv V50M. However, the average urinary concentrations of other mutations, such as the following: patient one, Ser75pro; patient three, Tyr134Cys; patient one, Ile127Val; and patient two, er70Ile. When measured by the same methods these values were 145 ± 45 μg/g·Cr, suggesting that our findings on urinary TTR concentrations in the ATTRv Val50Met patients should represent the general findings in most ATTRv patients. 

Although the creatinine, β_2_M, and NAG concentrations, which reflect kidney function, were not significantly correlated with the urinary TTR concentration (in creatinine: *r* = 0.358, *p* = 0.110, β_2_M: *r* = 0.215, *p* < 0.375, NAG: *r* = 0.087, and *p* < 0.720), TTR, and albumin correlated with the Kumamoto ATTRv score. In a comparison of the correlation between the TTR concentrations versus the Kumamoto ATTRv score, and the albumin concentrations versus the ATTRv score, the TTR concentrations showed a much stronger correlation than that of the albumin concentrations (Figure 2D,E). These results suggest that TTR concentrations in urine may become a more sensitive biomarker of ATTRv V50M progression than albumin [21,22].

To evaluate the presence of variant TTRs and the post-translational modification of urinary TTR, MS was utilised, as described in the Patients and Methods section [18,19,23]. As Figure 4 demonstrates, TTRv, in addition to TTRwt, was detected in patients with ATTRv V50M amyloidosis, and the cysteine-conjugated form of the TTR-oxidized form was predominant. These results suggest that TTR underwent oxidation during the process of its urinary excretion. As previous reports have described, oxidized TTR may play an important role in the amyloid formation mechanism [24,25,26].

As demonstrated in the Western blotting (lane 1 of Figure 3), TTR forms tetramers, in addition to a small proportion of monomers and dimers [14] in the blood, and tetrameric TTR is stable and does not easily dissociate into monomers in the blood of healthy subjects. Since the molecular mass of TTR in the tetrameric form is 55 kDa, and given that TTR binds to thyroxine and retinol-binding proteins [27], it does not readily undergo glomerular filtration in healthy subjects. In contrast, with regard to the amyloid fibril formation mechanism of ATTRv amyloidosis, the dissociation of TTR tetramers into TTR monomers has been well documented as a rate-limiting step to amyloid fibril formation [28].

In our study, why were urinary TTR concentrations correlated with the progression of ATTRv amyloidosis? To explain this correlation, we offer the following two possibilities: (1) The susceptibility of tetrameric TTR to dissociation may increase as the disease progresses. (2) As the disease progresses, so does the amyloid deposition in the kidneys, with the result being that the amount of urinary TTRs may increase. Both changes may lead to the aforementioned results.

## 5. Conclusions

The detection of TTR in urine may become a useful biomarker for ATTRv amyloidosis. The correlation of TTR concentrations in urine versus the ATTRv score, even in patients at early-stage ATTRv amyloidosis, is much stronger than that of the albumin concentrations and creatinine levels. Monomeric TTR in the urine may contribute to amyloid formation in the bladder as well as in other tissues and organs in the urinary tract.

## Figures and Tables

**Table 1:** Clinical information in healthy volunteers and ATTRv V50M patients.

**Healthy Subjects**															
**No.**	**Age (Years)**	**Gender**	**Serum TTR** **(mg/g Cr)**	**Serum Creatinine** **(mg/dL)**		**Urinary Albumin** **(mg/g Cr)**		**β** **2M (mg/g Cr)**		**NAG** **(IU/g Cr)**					
1	22	F	35.3	0.46		2.2		0.031		0.0					
2	22	M	22.8	0.58		3.1		0.054		1.871					
3	31	M	19.6	0.58		3.5		0.039		1.561					
4	24	F	24.3	0.59		4.1		0.063		1.532					
5	37	F	23.2	0.63		4.9		0.089		1.692					
6	28	M	28.4	0.70		5.4		0.031		3.043					
7	25	M	22.1	0.73		2.8		0.049		1.008					
8	26	F	19.3	0.76		5.9		0.030		1.492					
9	27	M	28.4	0.80		3.0		0.163		1.031					
10	27	F	25.6	0.80		8.0		0.073		2.793					
11	24	F	23.2	0.81		1.2		0.040		0.476					
12	34	F	24.9	0.89		4.1		0.057		1.131					
13	22	M	25.7	0.95		3.2		0.037		0.757					
14	34	M	29.3	0.95		6.6		0.058		2.623					
15	22	M	31.5	0.97		3.6		0.073		0.687					
**ATTRv V50M Amyloidosis Patients and Asymptomatic Carriers**															
**No.**	**Age** **(Years)**	**Gender**	**Serum** **TTR** **(mg/g Cr)**	**Serum** **Creatinine** **(mg/dL)**	**Urinary** **Albumin** **(mg/g Cr)**	**β** **2M** **(mg/g Cr)**	**NAG** **(IU/g Cr)**	**Liver-** **Transplant**	**ATTRv** **Score**	**Age at** **Disease** **Onset**	**Duration** **of** **Disease**	**Age at** **Liver-** **Transplant**	**Cardiac**	**Neurologic**	**L/E**
1	31	F	19.4	0.52	10.5	0.211	3.2	Yes	7	30	1	31	-	+	E
2	41	F	21.2	0.59	0.0	0.213	0.8	Yes	8	34	1	35	+	++	E
3	39	F	18.3	0.58	4.0	0.080	6.5	Yes	11	34	1	35	+	+	E
4	39	M	14.2	0.94	18.3	0.182	5.1	Yes	11	25	3	28	++	+++	E
5	28	M	24.3	0.84	0.0	0.139	5.9	Yes	11	27	1	28	-	+	E
6	50	F	15.3	0.61	204.0	0.248	2.6	No	11	37	4	41	+++	+++	E
7	42	M	14.1	0.62	13.5	0.127	2.3	Yes	16	28	4	32	+++	+++	E
8	37	F	18.1	0.5	6.7	0.225	2.5	Yes	18	29	2	31	+	++	E
9	37	F	19.2	0.67	6.8	0.254	3.6	Yes	22	30	3	33	+	++	E
10	36	M	17.3	0.84	9.1	0.112	2.2	Yes	23	28	2	30	++	++	E
11	36	M	15.8	1.07	57.6	0.203	5.8	Yes	26	28	2	30	+	++	E
12	42	M	16.1	0.47	49.0	0.062	1.6	Yes	26	38	3	41	+	+	E
13	50	F	13.2	0.79	4.8	0.164	4.1	Yes	14	37	4	41	+++	+++	E
14	72	M	17.7	0.69	183.7	0.241	2.9	No	55	65	-	-	+	++	L
15	63	F	16.9	0.56	3.9	0.216	4.2	No	44	59	-	-	+-	+	L
16	46	F	18.5	0.86	72.4	0.149	4.7	No	28	46	-	-	-	+	E
17	59	F	22.1	1.47	487.8	0.173	5.6	No	44	51	-	-	+	++	L
18	26	F	25.4	0.53	5.7	0.220	5.6	Carrier	-	-	-	-			
19	28	M	26.3	0.67	0.0	0.081	4.8	Carrier	-	-	-	-			
20	23	M	21.3	0.71	3.3	0.163	5.2	Carrier	-	-	-	-			
21	39	M	28.5	0.85	17.3	0.184	3.3	Carrier	-	-	-	-			

Cardiac—cardiac involvement, neurologic—neurologic impairment. L—late onset; E—early onset of ATTRv amyloidosis-: none, +: mild, ++: moderate, and +++: severe impairment of clinical manifestations.

## Data Availability

Human research data are not shared.
